# Medicine

**Published:** 1899-06

**Authors:** R. Shingleton Smith


### Progress of the Medical Sciences.

### MEDICINE.

In continuation of our former report (pp. 36-41) on the *rôle* of **milk in the production of tuberculosis**, a paper by Dr. Leonard G. Guthrie[1] is of interest. The following is a synopsis of his

[1] *Lancet*, 1899, i. 286.

conclusions:—1. Thoracic tuberculosis in children is more common than abdominal in the proportion of three to two. 2. Tabes mesenterica, as a cause of death in young children, is practically unknown. 3. The preponderance of thoracic over abdominal tuberculosis is not necessarily and solely due to the direct entry of bacilli into the air passages. In addition to this mode of infection, the lungs may be affected—(*a*) by bacilli entering the thoracic glands through the lymphatics of the pharynx, tonsils, and œsophagus above, and through the lymphatics of the intestines and the abdominal glands below; and (*b*) by the entry of bacilli through the thoracic ducts into the pulmonary circulation *viâ* the right heart. 4. Primary infection through the alimentary tract does not prove that food has been the sole source of evil; therefore tuberculosis in children is not likely to be materially checked by purification of the milk-supply alone. 5. The alleged increase of tuberculous meningitis of late years is probably due to pulmonary tuberculosis set up by severe epidemics of measles. Sims Woodhead[1] reports a large number of experiments in which it had been shown that milk had set up tuberculosis of the tonsils. It had been known for a very long time that pigs fed on tuberculous milk invariably developed a swelling under the jaw, but it was some time before attention had been drawn to the fact that apparently the invasion of the tubercle bacillus was through the tonsils. This mode of invasion had, perhaps, more to do with pulmonary tuberculosis than was sometimes imagined.

A year ago we directed attention to Gardiner's researches[2] on the **immunity from phthisis at high altitudes**, and from a perusal of such books as that of Solly[3] and of Weber,[4] we were almost led to the belief that high altitude is the one thing needful for the cure of tubercular diseases of the lungs; but now all this is changed. *The Practitioner's* new crusade (June, 1898) has been taken up so vigorously that now **sanatorium treatment** is everything, and altitude is nothing. Allusion was made to this matter recently,[5] but I make no excuse for again recurring to the topic, in which at present the whole world is keenly interested.

A publication of the views of a non-medical Nordrach patient, in the *Nineteenth Century*,[6] has been followed by a reply from Dr. Sinclair Coghill,[7] and again by a rejoinder from Mr. James Arthur Gibson.[8] These have been widely read, and all the world now inclines to the belief that medicinal treatment and climatic treatment are nothing in comparison with the methods associated with what is known as sanatorium treatment. These have been summarised in an excellent monograph,[9] and

[1] *Brit. M. J.*, 1899, i. 1038. [2] *Bristol M.-Chir. J.*, 1898, xvi. 127.
[3] *Medical Climatology*, 1897. (See p. 145 of this number.)
[4] *The Mineral Waters and Health Resorts of Europe*, 1898. (See p. 146 of this number.) [5] *Bristol M.-Chir. J.*, 1898, xvi. 235.
[6] 1899, xlv. 92. [7] *Ibid.*, 304. [8] *Ibid.*, 389.
[9] *Sanatoria for Consumptives*. By F. R. Walters. 1899.

here we find an account of some forty or more sanatoria, in various parts of the world, in which more or less successful results have been obtained at various altitudes, and some of them at comparatively low levels.

The essential and one common factor of treatment in all these results is the **open-air treatment**, and this has been well described by Dr. Burton-Fanning.[1] He admits that there is nothing new in the idea that a large percentage of phthisis cases should be curable, nor is there anything new in the vaunting of an open-air life for consumptives; but he adds, "We have failed in our management of phthisical cases through lack of insistance and perseverance." It is claimed that the elevation of the method into a complete system has become needful, and that the essential feature of the "cure" is the perpetual existence of the patient in the open air. "The whole *raison d'être* of these establishments is the practice of this particular cure, and not only do patients go prepared to submit themselves to it, but the whole spirit of the place and its equipment make the adoption of the routine an easy matter. The same broad principles govern the treatment at all these Continental sanatoria for consumption. . . . The *rationale* of this treatment lies in the removal of the patient from those conditions which favour the activity of the tubercle bacillus, while at the same time his constitutional resisting powers are sought to be increased in every conceivable way, to the end that he may successfully repel the attacks of his destroyer. . . . Seeing that the greater number of sufferers from consumption cannot avail themselves of the treatment which is offered abroad, and that in the case of those more fortunately situated banishment to a foreign land often involves many unnecessary hardships, the necessity for establishing sanatoria at home is manifest."

A writer in the *Medical Magazine* (April, 1899), on the *régime* of the Victoria Hospital for Consumption, Edinburgh, remarks: "The usual type of a tuberculous case is quite modified by open-air treatment. In an uncomplicated case the temperature remains subnormal. Night sweating is absent, cough is absent, dyspepsia is absent. Dr. Philip explains the usual cause of cough to be **irritation from vitiated air.** The same cause is to be assigned to the night sweating, $CO_2$ stimulating the sweating centre. It seems a very rational treatment to avoid the presence of the $CO_2$ in the air by plenty of fresh air in the room, rather than to be obliged to give a respiratory stimulant such as the usual picrotoxine, to get rid of the $CO_2$ in the body. Instead of having *dyspepsia*, patients begin to lay on weight rapidly. One patient had been in but three days who had already gained 1½ lb. In connection with weight, two types of patient were shown—the thin patient who put on flesh rapidly, and the fat, flabby patient who did not gain weight, but whose muscles

[1] *The Year-Book of Treatment*, 1899, p. 55.

began to feel firmer to the touch. The gaining weight was the good symptom in the former, the firming up the good symptom in the latter."

Dr. Pott describes the usual routine as follows[1]:—A patient comes for treatment. He has come shivering from his fireside, where he has been treated like an exotic, subject to chills and draughts, and breathing bad air. The first thing to do is to acclimatise him to live in a room in which there is a brisk fire, but in which the windows are gradually opened wider and wider, until at length he is accustomed to them being wide open all day. Having arrived at this stage, a fine day is chosen, and the patient, warmly wrapped up, is taken out into the balcony, and lies on a couch in a horizontal position, sheltered from the wind and rain, and basking in the sunshine. He is kept warm, plentifully fed with stimulating food, and provided with light occupation and congenial society. At first the period in the open-air is short, but it is lengthened until the patient is quite accustomed to spending the whole day in that way. The bedrooms are light, roomy, and well ventilated, and numerous well-thought-of ideas for the thorough continuance of the treatment and care of the patient are carried out under constant supervision. Breakfast is taken with open windows, and there are no special restraints or fussy rules to vex the residents. Caps and overcoats are worn indoors if required, and in fact, to use Dr. Pott's own words, "The regulated daily life of each patient is ordered with a view to getting well and learning to keep well." "The objects of the treatment are to combat the wasting and fever of the malady by the use of an excess of pure air, hygiene, a suitable and very full diet, regulated exercise, and rest."

It is admitted by Dr. Walters that "those who try to live in the open air in a climate like our own will meet with many difficulties, owing to the absence of special shelters and contrivances for warding off rain and wind while admitting fresh air." The credit of showing how this may be accomplished belongs mainly to Brehmer, Dettweiler, and their followers, and the essence of their methods is the elimination of haphazard treatment and the prescription of absolute repose or of various degrees of exercise, according to definite medical indications. "Patients who are febrile muts be kept at rest; if persistently febrile or with high temperatures at night, absolute rest in bed is needed, windows being kept open, or the bed wheeled on to the balcony according to weather and season and other indications."[2]

In spite of all the difficulties in carrying out open-air treatment in this country, Gibson says,[3] "As to a site for the sanatorium. Go to the highlands of Scotland, the lowlands of England, or to the bogs of Ireland, and plant your

[1] *Guardian*, January 21, 1899.

[2] Walters, *op. cit.*, p. 38. [3] *Loc. cit.*, p. 400.

sanatoria there—it is of little consequence where. I see many doctors, leaders of public thought on matters of health, still coquetting with climate. *Climate has nothing to do with the matter.* All that is *absolutely* necessary is (1) a spot in the country where *pure* air is to be had, (2) well away from smoke, dust, traffic, and excitement, where the patients may lead the quiet unconventional lives so necessary to their well-being; (3) the proper treatment, and (4) (but most important) the man to honestly carry it out. *These four things are indispensable*, nothing else is. . . . When it has been proved beyond doubt that consumption is quite as curable at home, on these lines, as it is abroad, it will be the duty of the State to undertake such measures as may be necessary for the cure, prevention, and final eradication of this disease." We fear that this time is yet far distant, and that the views of the enthusiast must as yet be accepted with some reservation. The diet in the sanatorium is one of the most essential elements of what Walther speaks of as the **proper treatment.** "*The movement in this country will succeed only in so far as it follows Nordrach lines*," says Gibson. All writers are agreed as to the necessity of good food, and a special kind of feeding is adopted at the different sanatoria on the Continent. The weight is usually taken every week regularly, and "if the patient does his duty, gains of from one to four pounds should be made weekly."

The method of forced feeding—the Debove method—was in frequent use some fifteen years ago, but now we hear little of it. This method is advised by Wilcox,[1] more especially for cases of tuberculous laryngitis. Wilcox recommends the separation of the meals into those containing the bulk of the starchy food and meals containing the bulk of the proteids. As a rule, the object at the various sanatoria is to get the consumptive to eat the maximum amount of nutritious and fattening food that can be tolerated, but the end is gained in slightly different ways at different places. The ingestion of this large quantity of food is helped by force of precept and influence, but the great promoter of appetite is fresh air.[2]

**The results of sanatorium treatment** appear from the published statements to be eminently satisfactory. They have been summarised by Walters,[3] but it is very difficult to make any comparison between institutions so variously situated. "Generally speaking, one may say that from one-fourth to one-third of the patients treated in sanatoria are practically cured, or a still greater proportion if they are treated in an early stage. Probably systematic and prolonged treatment from an early stage would restore to health from one-half to two-thirds of our consumptive patients, even without the advantage of an Alpine or other high altitude station. Unfortunately, it is quite out of the question to expect patients to submit to more

[1] *Med. News*, 1898, lxxii. 586.
[2] *Year-Book of Treatment*, 1899, p. 58. [3] *Op. cit.*, p. 49.

than a few months' treatment in a sanatorium, so that we must trust to the educational influence of the sanatorium to complete the recovery of those treated in it. . . . The good results of treatment are permanent in a large proportion of cases."

Gibson claims for Nordrach that the type of cases taken comprising all stages and including many failures from other sanatoria, the percentage of cures amounts to 30, that of nearly cured 65, leaving a balance of only 5 per cent. not improved. . . . "Of the twenty-two cases that I have known who went out to Nordrach from this country within the last four years, twenty were cured of consumption, one died from another cause, and the remaining one might have been cured if an operation on the lungs had been consented to by the patient's friends. . . . I have erred, I think, on the safe side in saying that Walther cures 90 per cent. of his cases."[1] These are the words of a grateful enthusiast who has been cured of his disease, but it yet remains to be demonstrated whether results like this can be achieved under any conditions in the climate of the British Islands.

In spite of the generally accepted view that the **notification** of phthisis is impracticable, a paper by Dr. Newsholme[2] points out that, so far as the patient is concerned, notification need imply, and ought to imply, nothing beyond sending him a precautionary circular, and that small difficulties of detail need not embarrass us. "If notification is desirable in the interests of the public health it should be adopted and a desirable principle is never impracticable." Hitherto the Local Government Board has not been convinced, and hence there is no need as yet to determine what limits to official action after notification are desirable.

R. Shingleton Smith.

[1] *Loc. cit.*, p. 396. [2] *Lancet*, 1899, i. 279.

